# Female Fertility and the Nutritional Approach: The Most Essential Aspects

**DOI:** 10.1093/advances/nmab068

**Published:** 2021-06-17

**Authors:** Kinga Skoracka, Alicja Ewa Ratajczak, Anna Maria Rychter, Agnieszka Dobrowolska, Iwona Krela-Kaźmierczak

**Affiliations:** Department of Gastroenterology, Dietetics and Internal Diseases, Poznan University of Medical Sciences, the Heliodor Swiecicki Hospital, Poznan, Poland; Department of Gastroenterology, Dietetics and Internal Diseases, Poznan University of Medical Sciences, the Heliodor Swiecicki Hospital, Poznan, Poland; Department of Gastroenterology, Dietetics and Internal Diseases, Poznan University of Medical Sciences, the Heliodor Swiecicki Hospital, Poznan, Poland; Department of Gastroenterology, Dietetics and Internal Diseases, Poznan University of Medical Sciences, the Heliodor Swiecicki Hospital, Poznan, Poland; Department of Gastroenterology, Dietetics and Internal Diseases, Poznan University of Medical Sciences, the Heliodor Swiecicki Hospital, Poznan, Poland

**Keywords:** diet, female fertility, nutrition, preconception, supplementation

## Abstract

Infertility is an increasing problem that affects couples attempting pregnancy. A growing body of evidence points to a link between diet and female fertility. In fact, data show that a diet high in *trans* fats, refined carbohydrates, and added sugars can negatively affect fertility. Conversely, a diet based on the Mediterranean dietary patterns, i.e., rich in dietary fiber, omega-3 (ɷ-3) fatty acids, plant-based protein, and vitamins and minerals, has a positive impact on female fertility. An unhealthy diet can disrupt microbiota composition, and it is worth investigating whether the composition of the gut microbiota correlates with the frequency of infertility. There is a lack of evidence to exclude gluten from the diet of every woman trying to become pregnant in the absence of celiac disease. Furthermore, there are no data concerning adverse effects of alcohol on female fertility, and caffeine consumption in the recommended amounts also does not seem to affect fertility. On the other hand, phytoestrogens presumably have a positive influence on female fertility. Nevertheless, there are many unanswered questions with regard to supplementation in order to enhance fertility. It has been established that women of childbearing age should supplement folic acid. Moreover, most people experience vitamin D and iodine deficiency; thus, it is vital to control their blood concentrations and consider supplementation if necessary. Therefore, since diet and lifestyle seem to be significant factors influencing fertility, it is valid to expand knowledge in this area.

## Teaser

**Statement of Significance**: This manuscript provides the current knowledge and a holistic view of diet and supplementation with regard to female fertility.

## Introduction

Infertility—a failure to achieve pregnancy after 12 mo of unprotected and routine sexual intercourse—affects many reproductive-aged couples attempting pregnancy (1, 2). It is estimated that ∼15% of couples worldwide experience difficulty becoming pregnant; however, female infertility contributes to only 35% of overall infertility cases, 20% of cases are related to both women and men, 30% involve problems only on the part of men, whereas 15% of infertility cases remain unexplained (3, 4). According to the WHO, infertility may affect ∼80 million women worldwide (5). Female infertility is defined as infertility caused primarily by female factors, such as ovulation derangements, reduced ovarian reserve, reproductive system disorders, or chronic diseases. Primary female infertility is diagnosed in women who have never borne a child. Secondary female infertility affects women who have given birth to a live child or who experienced a miscarriage but who simultaneously are unable to establish clinical pregnancy (6). Key definitions are provided in **Table 1**. Besides physiological, age-related factors, female fertility is also affected by the conditions related to the pathophysiology of the reproductive organs and several other factors, such as the environment and lifestyle. Endometriosis, deregulated ovarian functions, tubal infections, and cervical and uterine factors constitute the most common reproductive pathologies; however, the etiology of some female infertility cases remains unknown (7, 8).

**TABLE 1:** Definitions of essential terms according to the International Glossary on Infertility and Fertility Care, 2017 (6)

Term	Definition
Fertility	The capacity to establish a clinical pregnancy.
Infertility	A disease characterized by the failure to establish a clinical pregnancy following 12 mo of regular, unprotected sexual intercourse, or due to an impairment of a person's capacity to reproduce, either as an individual or with his/her partner.
Female infertility	Infertility caused primarily by the female factors encompassing the following: ovulatory disturbances; diminished ovarian reserve; anatomical, endocrine, genetic, functional, or immunological abnormalities of the reproductive system; chronic illness; and sexual conditions incompatible with coitus.
Male infertility	Infertility caused primarily by male factors encompassing the following: abnormal semen parameters or function; anatomical, endocrine, genetic, functional, or immunological abnormalities of the reproductive system; chronic illness; and sexual conditions incompatible with the ability to deposit semen in the vagina.

There is growing interest in lifestyle (including diet and physical activity), psychological stress, socioeconomic factors, BMI, smoking, alcohol, caffeine, and psychoactive substances in the context of fertility (9). Lifestyle—including caloric intake and diet composition in terms of vitamins, protein, lipids, carbohydrates, as well as the mineral content—seems to be especially vital in the context of infertility caused by endometriosis and ovulation disorders (9–12). Interestingly, the frequency and intensity of physical activity may differently affect fertility—intensive sports, influencing the hypothalamus-pituitary axis, may lead to hypothalamic amenorrhea and subsequently lead to infertility. However, moderate physical activity is recommended to improve ovarian function and fertility, especially among women with obesity or unable to handle stressful situations (11, 13). Moreover, many studies are currently investigating the association between the intestinal microbiota and female fertility.

In view of the abovementioned factors, it is vital to adopt a holistic approach to infertility treatment in both women and men, including many specialists (e.g., physicians, dietitians, physiologists, physiotherapists). In our nonsystematic review, we aimed to summarize the current knowledge regarding dietary aspects in female infertility. However, due to a lack of clear outcomes and the small number of intervention studies, we could not formulate dietary recommendations for reproductive-aged women planning a pregnancy. Our paper does not address the topic of diet and male infertility, although we emphasize that it is crucial to focus on the lifestyle and dietary factors in male infertility treatment, especially with regard to sperm quality. We devoted a separate paper to this area including a wide range of both topics (14).

## Current Status of Knowledge

We performed a literature search of MEDLINE (PubMed) searching for terms such as the following: fertility, fertility diet, female fertility, PCOS, endometriosis, infertility, infertility treatment. Since our paper is a narrative, not a systematic review, we may not have included all studies, and we must acknowledge a certain publication bias. However, every author of this publication conducted the literature search independently.

### Dietary habits and female fertility

Many researchers still investigate the influence of diet on fertility. Although there is undoubtedly an association between dietary habits and fertility, many questions remain unanswered. An individual diet, which comprises other comorbidities and lifestyle, is especially essential (15). In this section, we compared 2 different nutritional approaches which differently affect both female and male fertility.

#### The Mediterranean diet

As current studies indicate, a diet based on the Mediterranean diet (MeD) recommendations positively affects mental and physical health. The MeD has also been associated with favorable changes in insulin resistance, metabolic disturbances, and the risk of obesity, which is crucial in the context of fertility (5, 15). The MeD is characterized by a high consumption of vegetables (including pulses), fruits, olive oil, unrefined carbohydrates, low-fat dairy and poultry, oily fish, and red wine, with a low consumption of red meat and simple sugars (16).

In a review summarizing the main findings of a prospective cohort including 22,786 participants with a mean age of 35 y, a positive association between adherence to the MeD and fertility was suggested (16). Moreover, studies show that healthy dietary patterns can also increase the chances of live birth among women using assisted reproductive technology (ART) (17, 18). In a large cohort study by Chavarro et al. (19) in 17,544 women planning a pregnancy or who became pregnant during the study, there was an association between adherence to the pro-fertility diet (similar to the MeD) and a lower risk of infertility caused by ovulation disorders. The pro-fertility diet was characterized by a lower consumption of *trans*-fatty acids (TFAs) and a higher consumption of MUFAs and plant-derived protein, and decreased consumption of animal protein, low glycemic index foods, high-fiber foods, and—interestingly—high-fat dairy. Women following the pro-fertility diet consumed more nonheme iron and more frequently, i.e., at least 3 times/wk, took multivitamins, in particular group B vitamins (e.g., folic acid), consumed more coffee and alcohol, and were more physically active.

Kermack et al. (20) reported that supplementation of omega-3, vitamin D, and olive oil, which imitated the MeD, before in vitro fertilization did not affect the rate of embryo cleavage. The MeD correlated with RBC folate and serum vitamin B-6. Additionally, higher adherence to the MeD by couples undergoing in vitro fertilization increased the probability of pregnancy (21). It should be noted that a part of the MeD is moderate wine drinking and, for women, this equals 1 glass of red wine daily, although it may be quite controversial in the context of female fertility. We explain what impact alcohol consumption has on fertility later in this article. However, while the majority of research studies indicate dose-dependent relations between fertility and alcohol consumption, it should be taken into account that a number of pregnancies remain unplanned. Nonetheless, there are evidence-based recommendations to exclude alcohol from the diet of pregnant women (22).

#### The Western-style diet

In contrast to the MeD, the Western-style diet (WsD) is rich in refined and simple carbohydrates (mostly sugar, sweets, and sweetened beverages) and red and processed meat. Moreover, it is characterized by a low intake of fresh fruits and vegetables, unrefined grains, low-fat poultry, and fish. It could also be described according to its high caloric, fat, and high glycemic index intake, with a low consumption of dietary fiber and vitamins (23, 24).

According to the conducted studies, the WsD decreased IL-1RA concentrations and the cortisol-cortisone ratio in the follicular fluid, and reduced the number of blastocysts (25). Moreover, a higher consumption of fast food and a lower intake of fruit were associated with infertility, and with a moderate increase in the time to become pregnant (26). Additionally, an animal study indicated that the WsD altered ovarian cycles and affected hormone concentrations, decreasing progesterone and anti-Müllerian hormone. The study also demonstrated that the WsD increased the number of antral follicles and delayed the time to the estradiol surge (27).

It has been shown that a diet with a high glycemic index and rich in animal protein, TFAs, and SFAs may negatively affect fertility (5). These aspects will be discussed later in the paper. However, it should be noted that studies investigating the direct relation between the WsD and fertility are still necessary. A comparison between the MeD and the WsD with regard to female fertility is presented in **Table 2**.

### Dietary compounds and female fertility

#### Carbohydrates

Both insulin sensitivity and glucose metabolism can significantly affect ovulation and female fertility. In terms of carbohydrates, glycemic index and load are especially essential. Possibly, the consumption of high glycemic index products can increase insulin resistance, dyslipidemia, and oxidative stress, which negatively affects fertility and the ovarian functions (15, 33).

**TABLE 2:** Characteristics of the Mediterranean and the Western-style diets, and their influence on female fertility^1^

	Diet characteristics							
	Source of fat or fat type	Meat and fish	Dairy	Grains and legumes	Fruits and vegetables	Other	Influence on fertility	References
Mediterranean diet	MUFAs and PUFAs from nuts and olive oil	Poultry, moderate fish consumption	High consumption	Whole grain cereals, high consumption of legumes	Mostly fresh vegetables and fruits, high intake of dietary fiber	Moderate consumption of red, dry wine, low consumption of sweets	Direct: Increases chances of fertilization, supports ART	(17–19, 28–32)
Western-style diet	SFAs and TFAs from processed foods, meat, and fast-food	Red meat, processed meat	Low consumption	Refined cereals, low consumption of legumes	Low intake of fresh fruits, vegetables, and dietary fiber	High consumption of sweets and sweetened beverages	Indirect: Increases the risk of IR, T2D, and PCOS; impairs ovulation	(5, 23, 24)

^1^ART, assisted reproductive technology; IR, insulin resistance; PCOS, polycystic ovary syndrome; T2D, type 2 diabetes; TFA, *trans*-fatty acid.

Insulin regulates metabolism but also reproductive functions; it can modulate ovarian steroidogenesis as well as hyperinsulinemia which are correlated positively with hyperandrogenism and ovulation disorders. Insulin is also the primary regulator of the production of sex hormone–binding globulin (SHGB) among women with polycystic ovary syndrome (PCOS). High glycemic index and load have been associated with higher fasting glucose concentrations, hyperinsulinemia, and insulin resistance, and therefore with higher concentrations of insulin-like growth factor I (IGF-I) and androgens, which can lead to endocrine disturbances and, thus, may alter the maturation of oocytes (5). A large cohort study conducted in 18,555 women without a history of infertility, who planned or became pregnant during the study, showed that a higher consumption of carbohydrates at the cost of naturally occurring fats and with a high glycemic index was positively associated with infertility due to ovulation disorders (34). These results were confirmed by other studies where the higher consumption of high glycemic index products and carbohydrates, when compared with fiber intake, and a high consumption of simple sugars were related to lower chances of becoming pregnant (33). The main sources of added sugars are carbonated beverages, which can negatively affect fertility (35). Moreover, Machtinger et al. (36) observed that the consumption of sweetened, carbonated beverages—independently of the caffeine intake—can decrease the chances of reproductive success by means of ART. It has also been shown that the consumption of carbonated beverages is associated with increased concentrations of free estradiol (37).

Undoubtedly, both the amount and the type of carbohydrates are essential in the context of a pro-fertility diet among women with lipid and glucose metabolism disturbances. However, this aspect is also essential in the diet of reproductive-aged women planning to become pregnant.

#### Fat

Fats constitute a vital dietary compound affecting fertility. Hohos and Skaznik-Wikiel (38) suggested that a high-fat diet can be associated with changes in the reproductive functions, including menstrual cycle length, reproductive hormone concentrations [e.g., luteinizing hormone (LH)], and embryo quality in the ART cycles.

Furthermore, it seems that the quality of fat is more important than its amount. The Chavarro et al. study (39) comprising 18,555 women planning a pregnancy or who became pregnant during the study demonstrated that increasing the intake of TFAs by even 2% resulted in a significant increase in infertility risk due to ovulation disorders. In contrast, Mumford et al. (40) did not observe associations between TFAs, SFAs, and the relative risk of anovulation in the BioCycle Study. It is worth bearing in mind that the Chavarro et al. study was conducted in the United States between 1991 and 1995, and the first cohort study indicating the harmfulness of TFAs appeared only in 1993 (41). On the other hand, the BioCycle Study was conducted between 2005 and 2007, when the United States already had mandatory labeling of the TFA content in foods containing ≥0.5 g TFAs/serving (42). Furthermore, in another study, the negative influence of TFA intake on fertility was observed among 1290 American women planning a pregnancy. However, this association was not observed among the Danish women and, as the authors suggested, may be associated with a low consumption of TFAs among this cohort due to the 2003 Danish law requiring a limit of TFAs in fats and oils to 2% of the total fatty acids (FAs) (42, 43).

TFAs have proinflammatory properties and may increase insulin resistance, increasing the risk of developing type 2 diabetes or other metabolic disturbances, including PCOS, which can negatively affect fertility (39, 44–47). It has been assumed that the direct negative effect of TFAs is associated with their influence on and a decreased expression of peroxisome proliferator–activated receptor γ (PPAR-γ). Moreover, the intake of TFAs was associated with the incidence of endometriosis (48). According to the Global Burden of Diseases Study, differences in TFA consumption between countries in 2010 range from 0.2% to 6.5% of energy intake, whereas the mean global TFA intake is 1.4% of the total energy intake (39). The highest intake of TFAs is observed in Egypt, Pakistan, Canada, Mexico, and Bahrain, although the WHO recommends limiting consumption of TFAs to <1% of total energy intake (40). Some countries, following the example of Denmark, have taken action to limit the amount of TFAs in food by introducing TFA limits in food or by compulsory labeling of products containing TFAs. It seems that prohibiting TFAs is the most effective approach to reduce the amount of TFAs in the food supply (49). In countries where there are no limits on the amount of TFAs in food, products high in TFAs can still be found in supermarkets and are often cheaper than their TFA-free counterparts. Therefore, it seems that it is necessary to continuously increase the nutritional awareness of the public, as well as to learn how to read labels in order to make proper nutritional choices (44).

On the other hand, ɷ-3 FAs can positively affect fertility, as they play an essential role in steroidogenesis and have significant anti-inflammatory properties (50, 51). Currently, the available studies indicate that ɷ-3 FAs from oily fish or supplements have a beneficial effect on the growth and maturation of oocytes, decrease the risk of anovulation, and improve embryo morphology, and are associated with higher concentrations of progesterone (40, 51, 52). However, the results of the association between ɷ-3 FAs and fertility are contradictory. In numerous studies, no association, or insufficient evidence, has been observed (39, 43, 53–56). It seems, however, that ɷ-3 FAs—by increasing insulin sensitivity and improving the lipid profile—may be helpful in the treatment of PCOS, although more studies are required (57). The supplementation of ɷ-3 FAs decreases follicle-stimulating hormone (FSH) among women with normal weight, which has not been observed in women with obesity. On the basis of this study, it is possible to suggest that ɷ-3 FAs extend the reproductive lifespan (58). Nevertheless, further investigations among women with a diminished ovarian reserve are critical. Nassan et al. (59) demonstrated that the consumption of fish, which is a good source of ɷ-3 FA, was associated with a higher probability of live birth following ART. On the other hand, according to the study by Stanhiser et al. (60), no association was observed between concentration of ɷ-3 FAs and the probability of becoming pregnant naturally. Additionally, the consumption of seafood increases sexual intercourse frequency and provides greater fecundity (61).

Conversely, MUFAs can bind with the PPAR-γ receptor, thus decreasing inflammation and positively affecting fertility. In fact, studies have presented a positive correlation between the consumption (62) and concentration in plasma (53) of MUFAs, fertility, and the time to achieve pregnancy.

Studies investigating the influence of dairy-derived fats on fertility are interesting, although the results are often contradictory. On the one hand, according to the study by Chavarro et al. (63), the consumption of low-fat dairy—including low-fat milk, yogurt, and cottage cheese—increased the risk of infertility due to anovulation, whereas high-fat dairy increased fertility. This may possibly be associated with a higher content of estrogen and fat-soluble vitamins in high-fat dairy. Moreover, it could also be assumed that the beneficial effect of dairy-derived fat may be associated with the presence of the *trans*-palmitoleic acid, which seems to improve insulin sensitivity (64, 65). On the other hand, Wise et al. (66) did not confirm that the consumption of high-fat dairy is correlated with increased fecundity, and they did confirm that consuming lactose and low-fat dairy did not negatively affect fertility.

It is vital to note that the consumption of >3 portions of dairy/d decreases the risk of endometriosis diagnosis by 18%, when compared with the consumption of 2 servings (67). Additionally, women consuming >4 portions of dairy daily during adolescence presented a 32% lower risk of endometriosis during adulthood than women consuming ≤1 portion (68). Moreover, the total dairy intake was positively associated with live birth among women aged ≥35 y (69).

Taking the abovementioned facts into consideration, a high consumption of MUFAs and PUFAs (including a high consumption of ɷ-3 from oily fish or from supplementation) with a low consumption of TFAs and SFAs should be recommended to childbearing-age women trying to become pregnant. Moreover, the evidence for a positive influence of reduced-fat dairy and an increased consumption of high-fat dairy is scarce; thus, it should not be recommended. However, more studies are necessary.

#### Protein

The next element of a fertility diet is protein. Chavarro et al. (70) suggested that animal protein consumption has been associated with a higher risk of infertility due to a lack of ovulation. In turn, the intake of plant protein increases fertility among women >32 y. The difference may stem from the disparate impact of plant and animal protein on insulin and IGF-I secretion. Insulin response is lower after plant protein consumption than following animal protein.

According to Mumford et al. (71), protein intake—in particular animal protein—correlated negatively with testosterone concentrations among healthy women. It seems that androgens, i.e., testosterone, play an important role in regulation of the ovarian function and female fertility. However, excessive androgen signaling seems to be a major factor in androgen-related reproductive disorders, since it disturbs the pathways regulating ovarian follicular dynamics (72). However, protein intake was not associated with estradiol, progesterone, LH, and FSH concentrations. Additionally, the study showed a lack of association between the total, plant, and animal (without protein from dairy products) protein intake and the amount of antral follicles among women experiencing infertility (71). On the other hand, a high protein intake from dairy products was connected with a decreased number of antral follicles, which is a biomarker predicting ovarian primordial follicle numbers (73).

Furthermore, increasing protein intake may improve carbohydrate-insulin balance, which seems to be important in treating infertility due to a lack of ovulation. It is vital to notice that protein presents the highest satiety properties, affects diet-induced thermogenesis, and protects muscle mass (74).

Future studies on protein's role in the diet of women attempting pregnancy are necessary. In fact, protein should be included in the diet in the amount recommended for the rest of the population, based on such elements as the level of physical activity. Additionally, the diet ought to contain especially plant protein sources.

#### Micronutrients

##### Folic acid and vitamins B-12 and B-6

It is possible that folic acid, vitamin B-12, and vitamin B-6 affect fertility. Studies indicate that the supplementation of folic acid (particularly in a dose higher than the recommended one for the prevention of congenital defects and combined with vitamin B-12) in the period prior to pregnancy may increase the chances of becoming pregnant and ART success. However, there is no randomized controlled trial on the impact of a high dose of folic acid associated with a positive response in observational studies (75).

In fact, fortification of cereals with folic acid increased the number of twin births in the United States. However, this possibly results from an increasing number of women using the ovulation-inducing drugs and not the increased folic acid intake (76). It is vital to note that folic acid supplementation has been negatively associated with a shorter length of the menstrual cycle (77). Murto et al. (78) showed that women with unexplained infertility supplemented more folic acid than fertile women. Additionally, women experiencing infertility had higher concentrations of folic acid and lower concentrations of homocysteine when compared with the control group. On the other hand, the intake of synthetic folic acid was associated with an increase in progesterone and a decreased risk of sporadic anovulation (79).

Additionally, women with methylenetetrahydrofolate reductase (MTHFR) mutation achieved a lower percentage of in vitro fertilizations than subjects without a mutation. On the other hand, the prevalence of implantation and clinical pregnancy was similar in both groups (80). Moreover, the concentration of vitamin B-12 and folic acid was not associated with in vitro fertilization probability (81).

The impact of folic acid, vitamin B-12, and vitamin B-6 on fertility is possibly associated with homocysteine metabolism. A lack of vitamin B-12 disturbs the remethylation process, whereas vitamin B-6 deficiency directly leads to an accumulation of homocysteine due to the induction of an enzyme called cystathione b-synthase. Consequently, the transsulfuration process, through which histamine is converted to cysteine, decelerates (82). Clinical studies show that hyperhomocysteinemia combined with a low concentration of folic acid constitutes a risk factor for recurrent miscarriage. Additionally, a higher homocysteine concentration has been associated with a faulty vascularity of chorion among women with a recurrent early pregnancy loss (83). In fact, it is homocysteine that induces trophoblast apoptosis and decreases chorionic gonadotropin (84), whereas a high concentration of homocysteine causes endothelial inflammation through increased expression of proinflammatory cytokines (85). Moreover, an increased homocysteine concentration in the ovarian follicle liquid may affect the interaction between the ovarian follicle and the spermatozoon, decreasing the chances of fertilization (86). Additionally, hyperhomocysteinemia increases oxidative stress, which affects women's fertility (87).

A cohort study including 259 women who were regularly menstruating and not using hormonal contraceptives and diet supplements showed a connection between a higher homocysteine concentration and an increased risk of a lack of ovulation by 33%. Furthermore, a higher folic acid to homocysteine ratio decreased the risk of anovulation by 10% (88). In fact, mild homocysteinemia is often observed in mothers of children with neural tube defects (89). It is vital to note that women experiencing PCOS present homocysteine metabolism disorders and a higher concentration of homocysteine in comparison to healthy women (90). The supplementation of folic acid is recommended for women with PCOS (91).

Future studies are necessary to confirm whether lowering homocysteine concentrations through diet or supplementation with folic acid and vitamins B-6 and B-12 may improve ovarian function in women attempting pregnancy.

According to the recommendations, women should supplement with folic acid in the period prior to pregnancy, since supplementation is safe and does not cause side effects. Nevertheless, there is a further need for randomized trials confirming the impact of folic acid supplementation on fertility, as well as in doses higher than recommended for preventing neural tube defects (88).

##### Vitamin D

Vitamin D likely participates in the modulation of female reproductive functions. Studies have demonstrated that vitamin D receptors are expressed in numerous tissues of the reproductive organs, such as ovaries, endometrium, placenta, pituitary gland, and hypothalamus (92–95). Additionally, vitamin D affects various endocrine processes and the steroidogenesis of sex hormones (96, 97). A study indicated that serum concentration of vitamin D may be associated with PCOS and endometriosis and affects the success of ART (98). On the other hand, there was no association between vitamin D and fertility among healthy subjects (99). The deficiency of vitamin D affects calcium balance, increases the production and secretion of proinflammatory cytokines, as well as participates in glucose metabolism through stimulating the synthesis and secretion of insulin. Therefore, many studies discuss the impact of vitamin D on inflammatory diseases, including diabetes and cardiovascular disease (100). Moreover, vitamin D may be an essential component of PCOS development by means of regulating glucose metabolism (92). In fact, insulin resistance and hyperinsulinemia are associated with enhanced androgen synthesis in the ovaries and a lower concentration of SHGB (101).

The meta-analysis by He et al. (102) showed a lack of significant differences in vitamin D concentration between women with PCOS and healthy individuals. Nevertheless, the authors emphasized a significantly varied prevalence of vitamin D deficiency among women with PCOS that was associated with comorbidities. In fact, women with PCOS with vitamin D deficiency more frequently presented endocrine and metabolic disorders than women with the normal vitamin D concentrations. It is vital to note that vitamin D has anti-inflammatory and immunomodulating properties, and its deficiency may be associated with endometriosis, which is one of the causes of infertility (103, 104). In vitro animal studies (105–108) showed that vitamin D has beneficial effects on endometrial tissues, although clinical studies on the role of vitamin D in the diagnosis and treatment of endometriosis provide inconclusive evidence (109–111).

Furthermore, in a meta-analysis, Chu et al. (112) suggest that there is an association between vitamin D status and ART results. Additionally, the authors highlighted that vitamin D deficiency may be an essential factor in infertility treatment using ART. On the other hand, Abadia et al. (113) reported that vitamin D may be linked to a higher rate of fertilization in women undergoing ART. Nevertheless, this was not associated with a higher probability of live birth, or pregnancy.

Future studies are necessary to assess the association between vitamin D and PCOS, endometriosis, and with women's fertility. It is vital to note that a deficiency of vitamin D is common. Individuals presenting too-low concentrations of this vitamin should supplement vitamin D in doses of ≥1500–2000 IU/d (114).

##### Minerals

The proper concentration of minerals is essential for many physiological processes, including maintaining the normal quality of oocytes and embryo fertilization, maturation, and implantation (115). A deficiency of minerals may disturb fertility; therefore, women should pay attention to the proper intake of minerals and supplement the elements that could be deficient. One study showed that many women fail to meet nutrient needs—particularly in terms of folic acid, calcium, iodine, iron, selenium, vitamin D, and vitamin B-12—and thus have lower blood concentrations (116). Calcium, iron, zinc, magnesium, iodine, and selenium are especially essential with regard to fertility.

Calcium affects blood vessels, muscle contractions, nerve conduction, and hormone secretion. Additionally, the fetus uses the mother's skeletal calcium for bone growth. Therefore, the recommended dose of calcium constitutes a crucial element in the diet of women of childbearing age (117). Additionally, calcium deficiency may decrease vitamin D concentrations and increase the risk of hypertension, and pre-eclampsia. However, no studies refer to the validity of the supplementation of or fortification with calcium in the period before pregnancy to prevent pregnancy complications (118, 119).

Few studies have reported on the association between serum iron concentration and fertility. However, both excess and deficiency of iron may negatively affect fertility (120). According to Hahn et al. (121), total or heme iron intake was poorly associated with fecundity, particularly among women with a potential risk of iron deficiency, e.g., women with frequent and heavy periods. On the other hand, a prospective study showed that the supplementation of total and nonheme iron may decrease the risk of infertility due to disorders of ovulation (122).

Another key element is iodine, affecting thyroid gland function, which is essential for proper fertility. In a study conducted in 501 women experiencing moderate or severe iodine deficiency, pregnancy was delayed, and the chances of becoming pregnant in each cycle decreased by 46% when compared with women who were not iodine deficient. Among women with mild iodine deficiency, this association was minimal (123, 124). It is vital to note that mild and moderate iodine deficiency is common among women of reproductive age around the world (125–127).

Grieger et al. (128) reported that low serum concentrations of zinc and selenium were associated with a 1-mo longer period before achieving pregnancy. Additionally, a deficiency of selenium and copper, but not zinc, was linked to a higher risk of infertility. On the basis of limited studies, the impact of zinc and copper concentration on women's fertility remains unclear, and future research is required (128).

Selenium also affects thyroid gland function. Additionally, it is an antioxidant participating in the reduction of oxidative stress. In fact, selenium possibly influences the growth and maturation of oocytes. Therefore, an adequate supply of selenium is necessary (129).

Magnesium takes part in glucose metabolism; hence, it may be vital for women with PCOS and metabolic disorders. The proper serum concentration of magnesium is probably associated with increased insulin sensitivity of tissues (130, 131).

A balanced and varied diet allows for covering the requirements of daily nutrients. However, supplementation is necessary in the case of mineral deficiency, especially with regard to iodine (132, 133).

##### Phytoestrogens

The impact of phytoestrogens on fertility has been a highly controversial topic for years. Phytoestrogens are compounds of plant origin, including isoflavones found in soy products; lignans found in nuts, seeds, and cruciferous vegetables; as well as coumestans found in sprouts, peas, and beans (104).

On one hand, numerous scientific studies indicate the preventive effect of phytoestrogen consumption on the development of breast and endometrial cancer, fibroids, osteoporosis, cardiovascular diseases, inflammation, metabolic syndrome, and obesity (104–109). In fact, soy isoflavone supplementation was associated with an increase in the number of live births following clomiphene therapy, increased endometrial thickness, pregnancy rates following insemination, and in vitro fertilization. Furthermore, soy consumption was associated with an increased chance of live birth using ART (134–137).

On the other hand, certain studies point to endocrine system disorders as negative effects of phytoestrogen consumption. In the Adventist Health Study, women who consumed a greater amount of isoflavones were at an increased risk of never becoming pregnant and being childless (138). In contrast, a cohort study by Mumford et al. (139) found no association between soy intake and fertility.

In an analysis of 2 cohorts comprising women planning a pregnancy in North America and Denmark, which included 4880 and 2898 women, respectively, no strong association was observed between dietary phytoestrogen intake and the chances of becoming pregnant (140). At the same time, it is worth considering that, in Western countries, the average intake of phytoestrogen is <2 mg, and in European countries, the intake is even lower than 1 mg compared with the ∼50 mg consumed in Asian countries (141).

Undoubtedly, phytoestrogens are still not fully understood; therefore, further research in this area is desirable (142).

##### Gluten

Among women struggling with infertility, a discussion of the negative influence of gluten on fertility is relatively common—for instance, the study by Harper and Bold (143) asked subjects about their motivations for eliminating gluten from their diet. However, according to the recommendations, the exclusion of gluten from the diet is not recommended for the general population, and there is no evidence that it is beneficial in non-celiac individuals (144).

Castaño et al. (145) conducted a meta-analysis that included a total of 23 research studies, and aimed to assess the prevalence of celiac disease seroprevalence in women with fertility disorders. The study group consisted of women with overall infertility, women with idiopathic infertility, and women with recurrent spontaneous abortions. The studies included in the meta-analysis did not comprise women with a diagnosed celiac disease or allergy to wheat proteins. The meta-analysis demonstrated that celiac disease seroprevalence among women with infertility amounted to ∼1.3–1.6%, which allows estimating that women experiencing such disorders are 3 times more likely to develop celiac disease. However, due to the small number of respondents, it is impossible to precisely calculate the total incidence of the association between celiac disease and fertility disorders.

There are no recommendations indicating the benefits of eliminating gluten from the diet of all women experiencing infertility. It should be noted that many research studies indicate a much lower nutritional value of gluten-free diets compared with traditional diets (146). Nevertheless, such frequent diagnoses of previously undiagnosed celiac disease among women experiencing infertility raises the question of whether it is not reasonable to conduct celiac disease screening tests in women with infertility (147). However, there can be no doubt that women diagnosed with celiac disease attempting pregnancy should follow a gluten-free diet (148).

##### Antioxidants

The current knowledge indicates that oxidative stress, i.e., the imbalance between reactive oxygen species (ROS) and antioxidants leading to cell damage, plays an essential role in the development of infertility (149–151).

It is assumed that cytochrome P450 is involved in the production of ROS, and oxidative stress subsequently promotes the development of endometriosis, hydrosalpinx, and PCOS. Importantly, oxidative stress has also been shown to be associated with idiopathic infertility, recurrent miscarriage, and pre-eclampsia (152–155).

It has been proven that ROS entering the ovum causes damage, which has an important impact on the fertilization process and its further success, as well as the entire process of embryogenesis, which constitutes the reason for a wider use of antioxidants in the treatment of infertility (152, 155). The possible mechanisms of their action include improving blood circulation in the endometrium, lowering sex hormone concentrations, increasing tissue insulin sensitivity, and affecting ovulation, prostaglandin synthesis, and steroidogenesis (155, 156).

The most common environmental causes that exacerbate oxidative stress comprise environmental pollutants, smoking, drug use, alcohol abuse, malnutrition, poor diet, and chronic diseases, including obesity and autoimmune diseases (156).

A Cochrane review (157) indicates that there is evidence based on very-low-quality research suggesting that women experiencing infertility may benefit from antioxidant supplementation. The researchers emphasize that the quality of the available studies is not good enough to establish the possible side effects of the antioxidant supplementation. However, it is worth briefly discussing the individual antioxidants and their potential impact on fertility.

It is worth noting that women with endometriosis have been shown to have a lower supply of vitamins A, C, and E, as well as copper and zinc, than healthy women without fertility disorders (158–160). In fact, a 4-mo-long supplementation of vitamins C and E resulted in a reduction in oxidative stress (158). Additionally, higher levels of oxidative stress markers and lower serum concentrations of vitamins C and E have been observed in women suffering from PCOS (161, 162).

Vitamin C, vitamin E, and vitamin A are among some of the most potent antioxidants. Vitamin C, which is present in high concentrations in the cytosol of the oocyte, is essential, as it participates in collagen synthesis, which is significant for the growth of the Graaf follicle, ovulation, and the luteal phase. Moreover, vitamin C also helps restore oxidized vitamin E and glutathione (155). The benefits of vitamin E supplementation include improved epithelial growth in the blood vessels and the endometrium.

Moreover, inositol supplementation may be essential, particularly in PCOS, due to its insulin sensitivity–enhancing and insulin response–modulating effects (163, 164). Furthermore, inositol derivatives are important secondary messengers of the gonadotropins LH and FSH. Inositol has been shown to regulate the menstrual cycle, improve ovulation, and favorably influence metabolic parameters in women with PCOS, although there is a lack of research evaluating its association with the chances of pregnancy, miscarriage, or the number of deliveries (165).

Additionally, l-carnitine appears to be an important antioxidant. Research studies indicate that its supplementation relieves disorders of the reproductive system, such as PCOS, endometriosis, or amenorrhea (166–168). The alleviating effect of l-carnitine on endometriosis may be due to its impact on the hormonal balance, decreased cytokine release, and apoptosis. By means of its effect on the hypothalamic-pituitary-gonadal axis, l-carnitine regulates the concentrations of gonadotropins and sex hormones and thus may be beneficial for the course of PCOS and the menstrual cycle (166). l-Carnitine also increases energy production by oocytes through B-oxidation, and is involved in combating oxidative stress (166, 169). Interestingly, the bioavailability of carnitine from food is much higher than from supplements (170). Sharkwy et al. (171) conducted research to compare the clinical and metabolic profiles between N-acetylcysteine (NAC) and l-carnitine among women with clomiphene citrate–resistant PCOS. The study demonstrated that both NAC and l-carnitine were effective in improving pregnancy and ovulation rates among women with clomiphene citrate–resistant PCOS. However, although NAC was superior in increasing insulin sensitivity, only l-carnitine improved the lipid profile. In contrast, a study by Behrouzi Lak et al. (172) indicates that, in patients with PCOS without clomiphene citrate resistance, NAC is ineffective in inducing or augmenting ovulation in the PCOS patients who are able to undergo intrauterine insemination and, according to the authors, it cannot be recommended as an adjuvant to clomiphene citrate in such patients.

### Gut microbiota

The composition of the diet also plays an essential role in shaping the intestinal microbiota. Dietary components can either directly impact the gut microbiota by promoting or inhibiting its growth, or indirectly by means of influencing metabolism and the immune system, which can also lead to changes in the gut microbiota composition (173).

Studies indicate that the consumption of a Western diet has been associated with an increase in *Bacteroides* phyla and *Ruminococcus*. On the other hand, a high-fat diet has been positively correlated with the amount of *Bacteroides* and *Actinobacteria* simultaneously decreasing *Firmicutes* and *Proteobacteria*, which are positively correlated with the consumption of a high-fiber diet. Moreover, diets that are based on animal products have been associated with higher levels of *Alistipes, Bilophila*, and *Bacteroides* and with reduced levels of *Firmicutes*. In contrast, diets high in complex carbohydrates contribute to a beneficial increase in *Bifidobacteria*, with *Prevotella* being the most dominant bacterial type among vegetarians. The composition of the intestinal microbiota, largely dependent on diet, plays a vital role in the proper functioning of the immune system. Additionally, intestinal dysbiosis induces local inflammation and an increase in intestinal permeability, which is associated with a decrease in *Bifidobacteria*. These bacteria, in turn, can reduce LPS and improve the state of the intestinal barrier. All of the above-mentioned facts mean that the Western diet may, in fact, increase the risk of systemic inflammation (174, 175).

### Coffee and alcohol

A significant majority of research studies indicate that high caffeine consumption may constitute a potential factor associated with an increased time to achieving pregnancy and an increased risk of pregnancy loss (5, 7, 11, 176). In addition, a dose-dependent association has been observed between caffeine consumption during pregnancy and stillbirth, childhood acute leukemia, delayed fetal growth, and the negative effects on a child's birth weight, as well as on overweight and obesity in children (177, 178). According to the European Food Safety Authority, for pregnant women and for women attempting pregnancy, up to 200 mg of caffeine/d is recommended. Similarly, the American College of Obstetricians and Gynecologists indicates that the intake of up to 200 mg of caffeine does not appear to be a main factor leading to miscarriage or preterm delivery (179, 180). Nevertheless, in the latest review paper including 48 original observational studies and meta-analyses, James (178) emphasized that the assumptions about safe maternal caffeine consumption levels are not supported by the current evidence, and indicated a necessity for a radical revision of the current recommendations. Simultaneously, it is worth noting that the source of caffeine is not only coffee, but also tea, soft drinks, cocoa, or certain drugs (176).

On the other hand, there is evidence suggesting that alcohol consumption, especially heavy drinking and chronic alcohol consumption, has been connected to reduced fertility and a higher risk of developing menstrual disorders (22, 181). However, the mechanism in which excessive alcohol consumption negatively affects fertility has not been determined (5). A suggested hypothesis for the negative influence of alcohol intake on female fertility includes altering endogenous hormone concentrations, a direct impact on the maturation of the ovum, ovulation, early blastocyst development, and implantation (181). It is also crucial to stress that alcohol consumption during pregnancy can result in adverse effects in offspring development, such as fetal alcohol spectrum disorders (182).

## Summary of the Data Regarding a Diet for Women Planning a Pregnancy

Diet and nutritional patterns are undoubtedly significant for both male and female fertility; thus, it is worth investigating the components of the diet and their influence on fertility. Further research is needed to develop standardized dietary recommendations for women planning a pregnancy. The current knowledge on the effects of individual nutrients and their sources is summarized in **Table 3**. Further research is necessary to develop standardized dietary recommendations, which should be given to women planning a pregnancy, and individualized in case of problems with achieving pregnancy. It is important to emphasize the valid role of a clinical dietitian, who should actively participate in the care of women planning a pregnancy and, above all, be a member of a multidisciplinary team in infertility treatment centers.

**TABLE 3:** The effects of the selected dietary components on female fertility^1^

Nutrient	Summary	Recommended food sources	References
Carbohydrates	Added sugars and a high glycemic index have a negative effect on fertility.	Vegetables and fruit, whole-grain pasta, whole-grain bread, grains, rice, cereals	(5, 15, 33–35, 37)
Fat	Intake of TFAs and excess SFAs appears to negatively affect female fertility. The direct effect of PUFAs on fertility is unclear, while MUFAs appear to have a positive effect on female fertility.	Oily fish, rapeseed oil, flaxseed oil, olive oil and other plant oil, avocado, nuts, seeds	(39, 43, 53–56, 62)
Proteins	It is vital to include good sources of proteins in the diet. Plant proteins appear to have a positive impact on fertility, while animal protein—especially from processed meat—a negative impact.	Legumes, fish, lean meat, eggs, dairy products (particularly fermented)	(70, 71, 73)
Dairy	Studies regarding dairy are inconsistent—although dairy should be consumed as a part of healthy diet, it is hard to determine if it should be high-fat or low-fat in order to increase fertility. Taking current studies into the account, high-fat dairy should not be recommended in order to increase fertility, as it can have a negative impact on other risk factors for fertility.	Low-fat dairy, especially fermented dairy products	(63, 66)
Iodine	Iodine is essential for the proper development of the fetus and proper thyroid function. While mild and moderate iodine deficiency is common among women, it is crucial to pay special attention to the supply of iodine by women planning a pregnancy.	Iodized salt, dairy, seafood	(123, 125, 127)
Folic acid	It appears that folic acid supplementation, particularly combined with vitamin B-12, may increase the chance of pregnancy and ART success. There is a need for the randomized trials.	Green-leafy vegetables, eggs, poultry	(75, 81, 88)
Vitamin D	Serum vitamin D concentrations may be associated with PCOS and endometriosis and affect ART success. In a population of healthy, fertile individuals, there is no significant association.	Fish, eggs, cheese, milk, dairy	(98, 99, 112)
Antioxidants	Very-low-quality evidence suggests that antioxidant supplementation may be beneficial for women suffering from infertility. More research is needed to assess the risk of the possible side effects. Inositol, l-carnitine, and NAC require particular attention due to the increasing number of studies positively assessing their impact on parameters related to female fertility.	Fresh fruits (especially berry fruits) and vegetables, vegetable oil, spices (e.g., cinnamon), tea, coffee	(157)
Phytoestrogens	The relation of phytoestrogens to female fertility remains unclear. Studies indicate that the consumption of soy isoflavones has a beneficial effect on ART success.	Pulses, flaxseed oil	(134–137)
Gluten	In healthy individuals, gluten does not appear to affect fertility.	Not applicable	No research
Caffeine	High caffeine consumption may be a potential factor associated with the increased time to achieve pregnancy and an increased risk of pregnancy loss.	Coffee, cocoa—in recommended amounts	(5, 7, 11, 176)
Alcohol	There is some evidence suggesting that excessive alcohol consumption correlates positively with reduced fertility and a higher risk of developing menstrual disorders.	Not applicable	(22, 181)

^1^ART, assisted reproductive technology; NAC, N-acetylcysteine; PCOS, polycystic ovary syndrome; TFA, *trans*-fatty acid.

## Conclusions

Numerous questions remain unanswered, although there is no doubt that diet has an impact on female fertility. On the basis of the current knowledge, it can be confirmed that the consumption of TFAs, refined carbohydrates, and added sugars negatively affects female fertility. In contrast, a diet based on the recommendations of the MeD—rich in dietary fiber, ɷ-3 FAs, vegetable protein, vitamins, and minerals—has a positive effect on female fertility.

There are no clear guidelines on supplementation to enhance fertility in women. A properly balanced diet should provide all minerals and vitamins, except for vitamin D and folic acid, which should be supplemented. It may also be challenging to provide adequate amounts of iodine with the diet, especially in low-sodium diets and in elimination diets. Additionally, women in the period prior to pregnancy are also recommended to consume folic acid. Particularly in women considered as a risk group, serum concentrations of micronutrients and vitamins should be monitored, and in the case of deficiencies, supplementation should be introduced.

## Notes

The authors reported no funding received for this work.

Author disclosures: The authors report no conflicts of interest.

Abbreviations used: ART, assisted reproductive technology; FA, fatty acid; FSH, follicle-stimulating hormone; IGF-I, insulin-like growth factor I; LH, luteinizing hormone; MeD, Mediterranean diet; NAC, N-acetylcysteine; PCOS, polycystic ovary syndrome; PPAR-γ, peroxisome proliferator–activated receptor γ; ROS, reactive oxygen species; SHGB, sex hormone–binding globulin; TFA, *trans*-fatty acid; WsD, Western-style diet.
